# Is the female sex associated with an increased risk for long-term cognitive decline after the first-ever lacunar stroke? Prospective study on small vessel disease cohort

**DOI:** 10.3389/fneur.2022.1052401

**Published:** 2023-01-12

**Authors:** Aleksandra Pavlovic, Tatjana Pekmezovic, Milija Mijajlovic, Gordana Tomic, Jasna Zidverc Trajkovic

**Affiliations:** ^1^Faculty of Special Education and Rehabilitation, University of Belgrade, Belgrade, Serbia; ^2^Neurology Clinic, University Clinical Center of Serbia, Faculty of Medicine, University of Belgrade, Belgrade, Serbia; ^3^Faculty of Medicine, Institute of Epidemiology, University of Belgrade, Belgrade, Serbia

**Keywords:** small vessel disease (SVD), lacunar stroke, vascular cognitive impairment, white matter lesions, female sex

## Abstract

**Background:**

Sex is a significant determinant of survival and functional outcome after stroke. Long-term cognitive outcome after acute lacunar stroke in the context of sex differences has been rarely reported.

**Methods:**

A cohort of small vessel disease (SVD) patients presenting with first-ever acute lacunar stroke and normal cognitive status has been evaluated 4 years after the qualifying event for the presence of cognitive impairment (CI) with a comprehensive neuropsychological battery. Differences in baseline clinical and neuroimaging characteristics were compared between sexes in relation to cognitive status.

**Results:**

A total of 124 female and 150 male patients were analyzed. No difference was detected between the groups regarding age (*p* = 0.932) or frequency of common vascular risk factors (*p* > 0.1 for all). At the baseline assessment, women had more disabilities compared to men with a mean modified Rankin scale (mRS) score of 2.5 (1.5 in men, *p* < 0.0001). Scores of white matter hyperintensities (WMH) of presumed vascular origin and a total number of lacunes of presumed vascular origin on brain MRI were higher in women compared to men (*p* < 0.0001 for all). As many as 64.6% of patients had CI of any severity on follow-up, women more frequently (77.4%) than men (54.0%; *p* < 0.0001). Univariate logistic regression analysis showed that female sex, higher NIHSS and mRS scores, presence of depression, and increasing WMH severity were associated with an increased risk for CI. Multivariate regression analysis indicated that only depression (OR 1.74, 95%CI 1.25–2.44; *p* = 0.001) and WMH severity (OR 1.10, 95%CI 1.03–1.17; *p* = 0.004) were independently associated with the CI.

**Conclusion:**

At the long-term follow-up, women lacunar stroke survivors, compared to men, more frequently had CI in the presence of more severe vascular brain lesions, but this association was dependent on the occurrence of depression and severity of WMH, and could not be explained by differences in common vascular risk factors.

## Introduction

Due to the high survival and low recurrence rate, lacunar ischemic stroke is considered to have a favorable prognosis in the short term, with lower mortality, shorter hospital stays, and functional independence at discharge (1, 2). However, in the long-term follow-up, a history of previous lacunar stroke is associated with an increased risk for cardiovascular mortality, stroke recurrence, and functional and cognitive decline, particularly in older patients, those with multiple vascular risk factors, and initially more severe strokes (3, 4). The burden of underlying cerebral small vessel disease (SVD) worsens the long-term prognosis after lacunar stroke by increasing the risk of disability, rate of recurrence, the occurrence of cognitive impairment, and depression (5–7).

Sex is a significant determinant of long-term survival and functional outcome after stroke (8, 9). Stroke has a greater clinical effect on women, although age-specific incidence and mortality are higher among men (10, 11). Compared with men, women experience worse post-stroke functional and quality-of-life outcomes, which is partially explained by their older age at stroke onset and greater stroke severity (12).

Several studies reported that the female sex was an independent predictor of post-stroke cognitive impairment (CI) (13–17). The female sex has been also associated with both worse pre-stroke functioning and pre-stroke dementia (18, 19). In addition, women are more likely than men to experience post-stroke depression, which is a risk factor for cognitive decline, although the results are conflicting and age-related (20, 21). A variable proportion of patients after a lacunar stroke had been reported to have CI of any severity (mild cognitive impairment or dementia), ranging from 24 to 47%, with most of the studies referring to early post-event assessment, which may be influenced by acute complications and interventions and may even be reversible to a certain extent (4, 22). There is still a lack of knowledge regarding sex differences in post-stroke cognitive outcomes in the long term, as studies are few and heterogeneous in their designs (16, 19). Therefore, we aimed to investigate sex effect on long-term outcomes in patients with clinical and MRI evidence of cerebral SVD who initially presented with first-ever lacunar stroke.

## Methods

A cohort of consecutive patients with SVD hospital-treated for first-ever acute lacunar stroke and free of cognitive decline and depression was recruited and evaluated at the baseline, and then reevaluated 4 years after the qualifying event for the presence of CI in a single-center prospective study. Patients with pre-existing CI (*n* = 12), a severe motor deficit that precluded full neuropsychological assessment (*n* = 13), recurrent stroke during follow-up (*n* = 18), and lost for follow-up (*n* = 9) were not included in the final analysis. All patients underwent baseline brain MRI scanning per previously reported protocol (23, 24). Diagnosis of lacunar stroke was based on clinical and neuroimaging assessment. The functional, cognitive, and affective statuses were documented in all participants as described (23, 24). Standard neuropsychological testing was done in all patients at the baseline and at the follow-up, comprising the Mini-Mental State Examination (25), the Trail Making Test A and B (26), the Wisconsin Card Sorting Test (27), the Rey-Osterreith Complex Figure (28), the Rey Auditory Verbal Learning Test (29), the 60-item Boston Naming Test (30), and the Animal Naming Test (31). Standard age-, sex-, and/or education-adjusted norms were applied as appropriate. The neuropsychological battery is following the Neuropsychological Test Protocol proposed by the National Institute of Neurological Disorders and Stroke-Canadian Stroke Network Vascular Cognitive Decline Impairment Harmonization Standards (32). Instrumental Activities of Daily Living were documented in all patients (33). Only patients without recurrent stroke during the follow-up period were included in the study.

Baseline demographic data and vascular risk factors were compared between sexes. The admission NIH Stroke Scale (NIHSS) score was analyzed for all patients. Functional status was assessed using the modified Rankin Scale (mRS) score (23) at the recruitment. The patients with the lowest (0, *n* = 8) and highest (4, *n* = 12) mRS scores were excluded from the analysis to reduce study group heterogeneity in terms of stroke severity. During follow-up evaluation, patients were classified according to the findings of cognitive assessment as having: (1) normal cognitive status, (2) cognitive decline-no dementia, which included patients with evidence of a significant decline in at least one cognitive domain, but exclusion of dementia if impairment was not sufficiently severe to interfere with instrumental activities of daily living, or (3) vascular dementia with evidence of a significant decline in at least two cognitive domains and disturbance in instrumental activities of daily living, as reported before (23, 24). Data for patients with cognitive decline-no dementia and vascular dementia were jointly analyzed as CI. The presence of major depressive disorder was identified according to the DSM-IV criteria and patients were classified as depressed or not-depressed (34). The study has been approved by the Ethics Committee of the Clinical Center of Serbia and all patients consented to evaluation and follow-up.

## Magnetic resonance imaging and image analysis

Baseline brain MRI, performed on the 1.5-T scanner (Siemens Avanto, Germany), was evaluated by a trained neurologist blinded to clinical data with two visual rating scales of the severity of cerebral white matter hyperintensities (WMH) of presumed vascular origin on fluid-attenuated inversion recovery (FLAIR) and T2^*^-weighted axial scans (35–37). The total score on the Age-Related White Matter Changes (ARWMC) scale (tARWMC) was used as a measure of the whole brain (WMH) load (35). Basic ARWMC scores were rated on a four-point scale for brain regions, including frontal, parieto-occipital, temporal, basal ganglia, and infratentorial areas bilaterally, and were then summed to obtain the tARWMC (23, 35). Therefore, the tARWMC score was applied as a measure of the whole brain WMH and lacunes load, with the score ranging from 0 (no lesions) to a maximum of 30 (23, 35). In addition, the Fazekas visual rating scale was used for separate assessment of periventricular (PV) and deep subcortical (DS) WMH in all participants on FLAIR axial images, with scores ranging from 0 (no lesions) to a maximum of 3 (36). We identified lacunes of presumed vascular origin on FLAIR and T2 axial images as focal hyperintense lesions with a diameter of 3–15 mm (23, 36, 37), and included the total number of lacunes in the analysis.

## Statistical analysis

The normality of data distribution was tested by the Kolmogorov–Smirnov test, and data not normally distributed were presented as a median with an interquartile range. Statistical analysis comprised analysis of variance (ANOVA) for continuous variables and the Chi-squared test for categorical variables. In case of deviation from a normal distribution, a non-parametric Mann–Whitney U-test was used for testing differences between groups. Logistic regression analysis was used to compare data on clinical and MRI characteristics between sex groups. Multivariate logistic regression analysis was applied to identify independent parameters associated with the CI at follow-up, with risks shown as odds ratio (OR) estimated for each selected variable with a 95% confidence interval (95%CI). A value of *p* < 0.05 was considered statistically significant.

## Results

The group consisted of 124 female (45.3%) and 150 male (54.7%) patients, with a mean age of 62.8 ± 10.4 years. Male and female groups did not differ regarding age (*p* = 0.932) and education (*p* = 0.742, Table 1). No difference was found in the frequency of vascular risk factors (Table 1). Furthermore, the total number of vascular risk factors was the same between groups (*p* = 0.932) (Table 1). Only a minority of patients (*n* = 5) were on hormone-replacement therapy for menopause.

**Table 1:** Demographic data and vascular risk factor distribution in all patients.

	**Female (*n =* 124)**	**Male (*n =* 150)**	**All participants (*n =* 274)**	***p-*value**
Age (yrs.)	63.0 ± 11.0	62.6 ± 10.1	62.8 ± 10.4	0.932
Education (yrs.)	11.8 ± 2.0	11.9 ±2.4	11.9 ± 2.2	0.742
Hypertension	110 (88.7)	135 (90.0)	245 (89.4)	0.888
Diabetes mellitus	39 (31.4)	33 (22.0)	72 (26.3)	0.103
Hypercholesterolemia	101 (81.4)	127 (84.7)	228 (83.2)	0.584
Smoking	40 (32.2)	49 (32.7)	89 (32.5)	0.999
CAD	21 (16.9)	22 (14.7)	43 (15.7)	0.729
PAD	9 (7.3)	10 (6.7)	19 (6.9)	0.999
AF	10 (8.0)	13 (8.7)	23 (8.4)	0.999
Carotid stenosis >50%	10 (8.0)	9 (6.0)	19 (6.9)	0.663
Total number of risk factors	3.6 ± 1.4	3.5 ± 1.1	3.6 ± 1.2	0.932

Data are given as *n*, % or mean ± SD.

CAD, coronary artery disease; PAD, peripheral artery disease; AF, atrial fibrillation.

The clinical presentation did not differ between sexes (*p* = p.981), with most of the patients exhibiting pure motor stroke (76 or 61.2% in women vs. 99 or 66.0% in men), followed up by sensorimotor stroke (17 or 13.7% in women and 26 or 17.3% in men) and ataxic hemiparesis (12 or 9.7% in women and 13 or 8.7% in men), while the rest of the patients experienced a pure sensory stroke, dysarthria clumsy hand syndrome, and atypical lacunar syndrome. At the baseline, women had more severe strokes than men with an admission NIHSS score of 6.6 compared to 5.3 in men (*p* < 0.0001, Table 1). In addition, the mean mRS was also higher in women (2.5) compared to men (1.5) (*p* < 0.0001, Table 1). All measures of SVD-related lesions on MRI scans were more severe in female patients, including tARWMC score, Fazekas PV and DS scores, and the total number of lacunes (*p* < 0.0001 for all, Table 2).

**Table 2:** Baseline clinical and neuroimaging characteristics of patient subgroups.

	**Female (*n =* 124)**	**Male (*n =* 150)**	**All participants (*n =* 274)**	***p-*value**
NIHSS score, mean ± SD	6.6 ± 2.3	5.3 ± 2.3	5.9 ± 2.4	< 0.0001
mRS, mean ± SD	2.5 ± 0.5	1.5 ± 0.6	2.0 ± 0.8	< 0.0001
tARWMC scale score	15.9 ± 5.8	11.3 ± 4.8	13.4 ± 5.3	< 0.0001
Fazekas PV score	3 (0–3)	2 (0–3)	2 (0–3)	< 0.0001
Fazekas DS score	3 (1–3)	2 (0–3)	2 (0–3)	< 0.0001
Number of lacunes, total	10.8 ± 3.9	8.2 ± 3.6	9.4.±3.90	< 0.0001

Data are given as *n*, % or median (range) unless stated otherwise.

NIHSS, National Institutes of Health Stroke Scale; mRS, modified Rankin Scale; tARWMC, total Age-Related White Matter Changes; PV, periventricular; DS, deep subcortical.

**Figure 1:**
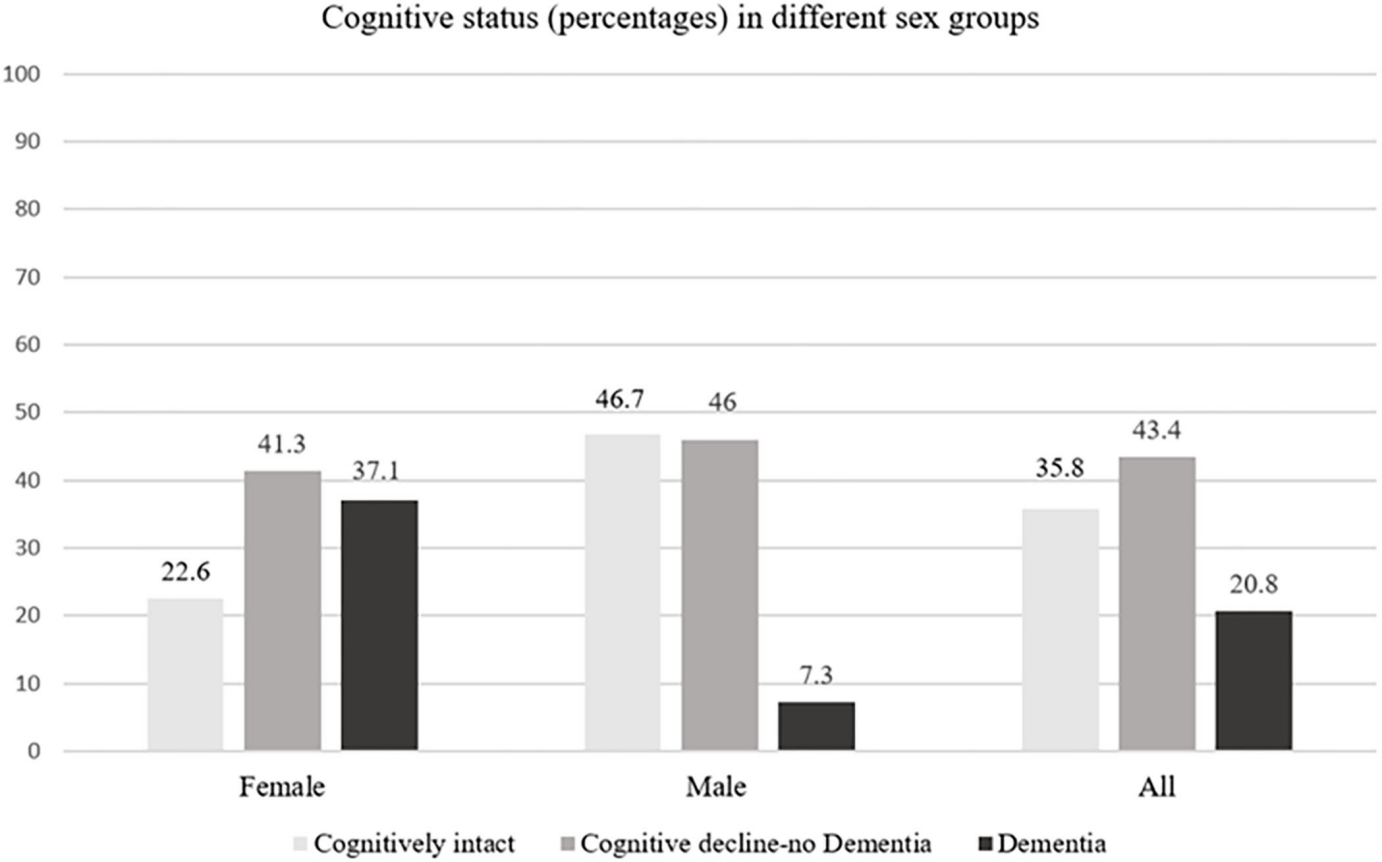
Distribution of cognitive status in female patients, male patients, and all patients with SVD.

A follow-up assessment was performed after a mean time of 47.4 ± 6.9 months, with no difference between sexes in the time of reassessment (*p* = 0.764). On follow-up, no difference in depression rate was detected between sexes (45 or 37.2% depressed female patients vs. 61 or 40.7% male patients, *p* = 0.791). Overall, any CI was detected in 177 or 64.6% of stroke survivors, more frequently in women than men (96 or 77.4% of female patients vs. 81 or 54.0% of male patients, *p* < 0.0001) (Figure 1). A total of 46 (37.1%) female patients met the criteria for diagnosis of dementia, and only 12 (8.0%) male patients (Figure 1). In patients with CI on follow-up, the most frequently affected cognitive domains were: attention (in 96 or all female and 76 or 93.8% male patients), processing speed (in 92 or 95.8% female and 78 or 96.3% of male patients), executive functions (in 86 or 89.6% female and 57 or 70.4% male patients), and visuospatial functions (in 57 or 59.4% female and 34 or 42.0% male patients), followed by memory (25 or 26.0% female and 5 or 6.2% male patients) and language (14 or 14.6% female and 1 or 1.2% male patients). The decline in global cognitive functioning was detected in 49 (51.0%) female patients and 9 (11.1%) male patients with CI.

Univariate regression analysis adjusted for age and vascular risk factors indicated that female sex, presence of depression, baseline total number of lacunes, and initial overall severity of WMH (tARWMC scale score, Fazekas PV, and Fazekas DS scores) were significantly associated with CI at follow-up (Table 3). Multivariate regression analysis adjusted for age and vascular risk factors revealed variables independently associated with the female sex in patients with SVD: depression with OR 1.74 (95%CI 1.25–2.44; *p* = 0.0001) and tARWMC with OR 1.30 (95%CI 1.03–1.17); *p* = 0.004 (Table 4).

**Table 3:** Variables associated with CI at follow-up; univariate logistic regression analysis adjusted for age and vascular risk factors.

	**OR (95% CI)**	***p-*value**
Female sex	1.44 (1.07–1.94)	0.015
mRS	1.14 (0.95–1.36)	0.163
NIHSS	1.02 (0.96–1.08)	0.591
Depression	2.09 (1.54–2.82)	< 0.0001
Number of lacunes	1.07 (1.04–1.11)	< 0.0001
tARWMC scale score	1.08 (1.01–1.0.16)	0.046
Fazekas PV score	1.34 1.17–1.53	< 0.0001
Fazekas DS score	1.67 (1.34–2.08)	< 0.0001

OR, odds ratio; mRS, modified Rankin Scale; tARWMC, total Age-Related White Matter Changes; PV, periventricular; DS, deep subcortical.

**Table 4:** Variables associated with CI at follow-up; multivariate logistic regression analysis adjusted for age and vascular risk factors.

	**OR (95% CI)**	***p-*value**
Depression	1.74 (1.25–2.44)	0.001
tARWMC scale score	1.10 (1.03–1.17)	0.004

OR, odds ratio, tARWMC, total Age-Related White Matter Changes.

## Discussion

In this prospective study of patients with first-ever lacunar stroke and imaging evidence of SVD but with normal cognitive status, more than half (64.6%) of surviving patients met the criteria for CI 4 years after the qualifying event, likely as a predictor of subcortical vascular dementia in the medium-long term. Patients with CI were more frequently women, with more severe baseline strokes, more disability after stroke, and more advanced cerebral SVD lesions according to the MRI-based scoring systems. At the baseline assessment, after the initial lacunar stroke in our cohort, sex differences were noted, with female patients having a more severe functional impairment and more extensive baseline MRI markers of SVD, including both WMH and the number of lacunes of presumed vascular origin. After a mean follow-up of 4 years, a significant difference in cognitive status between sexes was detected, with evidence for CI being significantly more frequent in women than men. These sex disparities could not be explained by differences in common vascular risk factors or age but were independently associated with the occurrence of depression and the burden of WMH of presumed vascular origin.

There is increasing evidence of sex-specific differences in stroke symptoms, diagnosis, treatment, and preventive strategies (10, 11, 38). Currently, specific recommendations for the prevention and management of stroke in women are available (39, 40). Although sex-specific differences in common vascular risk factors have been recognized, this was not observed in our cohort of patients nor was any particular risk factor associated with cognitive outcome (39, 41, 42). Other possible factors adding to the risk of SVD and cognitive outcomes, such as serum homocysteine levels, immunological or inflammation markers, blood pressure variability, and the influence of genetic factors, were not assessed in this study. Sex-hormone influence may be less relevant in this post-menopausal population (female patients' mean age was 62.5 years) and only a minority of patients were on the hormone-replacement treatment (40).

Cognitive impairment appears to be common after lacunar strokes despite their small size, suggesting that underlying SVD may increase their impact (4, 7, 43). Cerebral SVD burden has been strongly associated with post-stroke cognitive and functional outcomes (44, 45). Most studies have been focusing on the early post-stroke period but also indicating that women more frequently than men experienced cognitive deficits (16). In the Health and Retirement Study, Bako et al. detected a significant short-term acceleration of cognitive decline for the overall population (4.2 percentage points) and among female participants (5.0 percentage points), but no evidence of long-term acceleration of the cognitive decline after stroke was noted (16). We found a significant association between medium-to-long-term CI and the female sex, which is in accordance with studies reporting female sex was an independent predictor of post-stroke CI (13–15), although there are conflicting results as well (46–48). In their systematic review and meta-analysis, Pendlebury and Rothwell indicated that the female sex was confounded by age and was strongly associated rather with pre-stroke than with post-stroke dementia (18). In a prospective follow-up of 1,227 patients surviving a first-ever ischemic stroke or intracerebral hemorrhage, at 90 days after stroke, women had worse cognitive outcomes than men, with differences attributed to sociodemographic and pre-stroke characteristics (19). The rates of dementia were lower than in our study with 27.6% for men and 35.6% for women with an unadjusted odds ratio of dementia comparing women with men of 1.45, but the follow-up assessment in our study was done much later (19).

Andersen et al. also did not find evidence of a sex modifier for vascular dementia, in contrast to the female predominance of Alzheimer's type of dementia (49). Preexisting degenerative pathology may be more frequent in female stroke survivors developing cognitive impairment post-stroke (50), but none of our patients met the criteria for other types of dementia on follow-up assessment. Similarly, the increased burden of vascular changes attributable to SVD registered in our study in female patients compared to male participants led to an increased risk for CI. Interestingly, in our dataset of patients, the co-existence of depression was independently associated with the occurrence of CI post-lacunar stroke. Female stroke survivors may be facing additional challenges of post-stroke depression and unmet social needs, such as living alone or being a caregiver themselves before the stroke (19, 21, 24, 51–53). We were not able to determine the exact onset of depressive symptoms in our patients, and could not conclude whether depression preceded cognitive decline or developed as a parallel trajectory. Although cognitive trajectories post-stroke have been well-described, there is a need to increase our knowledge of affective status trajectories post-stroke, in patients with SVD in particular (54, 55). The presence of both cognitive and mood deterioration in patients with SVD may be indicative of a more severe vascular burden, as was the case in our study. Unfortunately, we did not control for other potential confounders, such as major life stressors and socioeconomic status (54, 55).

The impact of stroke subtypes on cognitive outcomes has been rarely studied, particularly in a long-term setting. Although lacunar strokes are considered a leading cause of vascular CI and vascular dementia, in a meta-analysis by Makin and co-workers, there were no differences in the proportion of patients with CI after lacunar and non-lacunar strokes (4). Corriani and co-workers examined the long-term risk of dementia among survivors of incident stroke of any type and recorded high rates of CI among survivors of hemorrhagic stroke comprising intracerebral and subarachnoid hemorrhage (56). In our study, all MRI markers of SVD were more severe in female than in male patients. This is in accordance with the Rotterdam Scan Study reporting tendency for women to have more severe WMH of presumed vascular origin (57) but also the more marked progression of subcortical WMH and incident lacunar infarcts compared with men (58). Similar were the findings of the Cardiovascular Health Study and the Atherosclerosis Risk in Communities Study (59, 60). Contrary to this, in a recent cross-sectional study, male sex was significantly and independently associated with the proposed total SVD score which combines individual MRI features of the SVD in one measure (61). A possible explanation for these sex differences was that postmenopausal estrogen reduction might make the female brain more vulnerable by reduction of cerebral blood flow and ischemia (62), but mechanisms are still largely unknown. In a community-based cohort followed up for 9 years, women with migraine had a higher incidence of deep white matter hyperintensities but did not have significantly higher progression of other MRI-measured brain changes (63). We did not analyze the data on the frequency of migraine in our patients or hormonal status. Overall, differences in common risk factors could not explain differences in WMH severity between sexes in our study.

Our female patients with SVD and lacunar stroke had on average mild to moderate disability compared to men who were significantly less disabled (mean mRS 2.5 in women, 1.5 in men). Most studies have indicated that stroke is more severe in women than in men, although this finding has been confounded by age (64–68). A subanalysis of the Framingham Heart Study showed that women were more disabled and 3.5 times as likely to be institutionalized at 3–6 months post-stroke compared with men, even in the first-ever stroke cases present in our study (69). After adjustment for cardiovascular risk profile, socioeconomic status, and age, stroke remained more severe in women in a study by Dehlendorff et al. (70). Lacunar stroke severity and outcome have been reported to be similar in men and women in some cohorts, while others documented a higher admission NIHSS in women compared to men (41, 68). The other possible explanation for the sex difference in functional outcome could also be related to the pre-stroke functional status, which appeared lower in women, and possibly differences in acute lacunar stroke location and volume which we did not analyze (68, 71). However, our findings are associated with a higher burden of pre-existing SVD in female patients compared to male patients with the cognitive outcome, which may be reflected in the functional score (mRS) as well.

There are several limitations to our study, comprising the size of the cohort, lack of monitoring for other imaging markers of SVD, such as cerebral microbleeds, perivascular spaces, and cerebral atrophy, single-center design, and engagement of a single though blinded MRI reader (72, 73). In addition, we did not analyze other confounding factors, such as the impact of the medications used by our patients or the development of new comorbidities, including the occurrence of new vascular risk factors during the follow-up period. However, we focused on a very well-defined cohort of a particular stroke subtype patients experiencing lacunar stroke and performed a close long-term follow-up to understand specific stroke subtype influence on cognitive outcome. Our findings have clinical implications of keeping in focus cognitive outcome even in long-term post-acute lacunar stroke to detect early susceptible patients, and in identifying a subgroup of post-lacunar stroke patients particularly prone to developing cognitive decline. The optimal management of post-stroke CI remains controversial both in general terms as well as regarding the sex differences in interventions for its prevention and treatment (51, 74).

## Conclusion

The female sex is associated with more severe vascular brain lesions secondary to SVD and cognitive deterioration long-term after acute lacunar stroke. The independent predictors of cognitive decline were the co-existence of depression and the severity of vascular WM changes. Mechanisms of these differences are beyond common vascular risk factors and are still to be explained. The cognitive outcome as well as affective status post-lacunar stroke should be actively monitored even in the long term, in women particularly, and could be a signal of more severe underlying SVD markers on neuroimaging.

## Data availability statement

The raw data supporting the conclusions of this article will be made available by the authors, without undue reservation.

## Ethics statement

The studies involving human participants were reviewed and approved by Ethics Committee of the Clinical Center of Serbia, Belgrade, Serbia. The patients/participants provided their written informed consent to participate in this study.

## Author contributions

AP designed the study, conducted research, and wrote the paper. TP performed the statistical analysis and contributed to the writing of the paper. MM, GT, and JZ contributed to the research and writing of the paper. All authors contributed to the article and approved the submitted version.
